# Using the Relative Entropy of Linguistic Complexity to Assess L2 Language Proficiency Development

**DOI:** 10.3390/e23081080

**Published:** 2021-08-20

**Authors:** Kun Sun, Rong Wang

**Affiliations:** 1 Department of Linguistics, University of Tübingen, 72074 Tübingen, Germany; 2 Institute of Natural Language Processing, University of Stuttgart, 70569 Stuttgart, Germany

**Keywords:** L2 learners, linguistic complexity, language proficiency development, information theory, time series

## Abstract

This study applies relative entropy in naturalistic large-scale corpus to calculate the difference among L2 (second language) learners at different levels. We chose lemma, token, POS-trigram, conjunction to represent lexicon and grammar to detect the patterns of language proficiency development among different L2 groups using relative entropy. The results show that information distribution discrimination regarding lexical and grammatical differences continues to increase from L2 learners at a lower level to those at a higher level. This result is consistent with the assumption that in the course of second language acquisition, L2 learners develop towards a more complex and diverse use of language. Meanwhile, this study uses the statistics method of *time series* to process the data on L2 differences yielded by traditional frequency-based methods processing the same L2 corpus to compare with the results of relative entropy. However, the results from the traditional methods rarely show regularity. As compared to the algorithms in traditional approaches, relative entropy performs much better in detecting L2 proficiency development. In this sense, we have developed an effective and practical algorithm for stably detecting and predicting the developments in L2 learners’ language proficiency.

## 1. Introduction

Measuring learners’ second language (L2) proficiency is an important issue not only as regards practical language teaching, but also with respect to research on L2 acquisition. To this end, in the following, both qualitative and quantitative methods have been employed, such as face-to-face interviews, standardized tests, and linguistic feature analysis and modeling. In recent years, a popular means of quantitatively measuring L2 proficiency has been to look at the linguistic features of the learners’ L2 production from the point of view of complexity [1,2], which is one aspect of the Complexity, Accuracy, and Fluency (CAF) framework for learner language analysis [3,4]. Complexity measures have been employed to gauge L2 proficiency and its development at multiple levels of linguistic representation such as lexis, morphosyntax, discourse, and psycholinguistics [5,6].

Linguistic complexity functions as a basic descriptor of L2 performance and as indicative of L2 proficiency and development in such research [1]. Hundreds of complexity measures have been developed and used in previous investigations [7,8,9]. In general terms, the calculation of these measures boils down to counting the number of linguistic components (e.g., words, dependent clauses, complex nominals, etc.) and the number and types of connections between these components [10]. Although research making use of these measures has helped to examine a number of important issues concerning L1 (the first language) and L2 acquisition, such as proficiency assessment, language development, and language teaching/learning [3,11,12], the methods for enumerating linguistic components and calculating their relative ratios make use of the available information. These studies contributed highly useful insights into the nature of L2 lexical/syntactic changes in general and of the development of L2 learners over time. Meanwhile, they also leave much room for further exploration. 

There are several potential limitations regarding these measures and methods. First, most of the studies that employed them were based on the summation or ratio algorithm of frequency. Despite the variety of measurement units that have become available, we have to admit that in practice the summation or ratio algorithm of frequency for the operation of these units has not been upgraded: still based on simple addition, subtraction and ratios of frequencies. Although *frequency* is a very important and useful measure in quantitative research, it is merely the sum of all the reasons why a word could occur often in a corpus. *Frequency* as a measure does not consider the context. The lexical and syntactic complexity measures used in the past studies are actually based on this algorithm of frequency summation or ratio. However, the development of computational algorithms allows us to develop methods that make more precise measurements. In order to reduce the limit of frequency, many studies attempted to use a diversity of methods to explore linguistic changes. For instance, the *n-gram* algorithm can be used to calculate the bi-/tri-/four-gram transitional probability, which considers the larger context or the probability of the context. 

Syntactic complexity, for example, employs measures such as dependent clause/T-unit (a T-unit is often a sentence), dependent clauses per clause, and complex nominals per clause to compute the ratios based on the frequencies of the respective components. The underlying algorithm used in these measures is the summation of frequency or the ratio of frequencies. However, these frequencies might not be comparable. This is because units (measures/metrics) with different meanings or usages are not exactly the same in different L2 corpora even though they might be categorized under the same label. A direct summation or ratio that is simply based on their frequency would completely ignore their contextual meanings or usages. For instance, “subordination” in English can be employed in a diversity of subordinate conjunctions that have different meanings and usages. That is to say, each subordinate conjunction (subordination) should be measured according to frequency by using different weights rather than by simply using frequency. However, the algorithm employed in past studies has ignored the distinctions between different types of subordination in meanings and usages. This can be illustrated using the following example. L2 learners at a lower level use a “that-clause” 10 times, while L2 learners at a higher level use various “time, contrast, reason-effect” adverbial clauses 10 times. An algorithm based on the summation or ratio of frequencies tends to treat the two cases as the same with regard to their use of subordination. This may be problematic because there is a variety of different kinds of subordination in the two groups. Similarly, the summation or ratio algorithm based on frequency that was used in the past studies also ignored the weights of the various lexical and grammatical units that were used to measure complexity and accuracy. This problem can be solved with a new algorithm that considers the weights of different units that were previously grouped under the same category. An alternative that is simpler and more effective is an algorithm that directly measures exactly the same linguistic units in two different groups of L2 learners. 

One of the assumptions in L2 developments research has been that learners use a more elaborate and diverse language as they progress in their development. Such progress could then be seen, for instance, in the use of longer sentences, more embedding, and a greater range of vocabulary and morphology [13,14]. More recently, this assumption has been challenged by an approach to linguistic complexity that, on the one hand, considers such complexity to be a multidimensional phenomenon [1,11,12] and, on the other hand, specifies the relation between complexity and proficiency more accurately [4]. The conflicting results from these studies could have been brought about by the use of different fine-grained measures. However, studies based on fine-grained measures of the development of L2 language proficiency still compared different units but without considering their different weights. In addition to this, the algorithm of summation (or ratio) of frequency in complexity was still applied in these fine-grained studies in order to assess L2 development. 

The present study proposes a practical and effective method for measuring the development of language proficiency in L2 learners. This practical and effective method should be capable of overcoming the aforementioned difficulties. The algorithm employed is that of relative entropy, which is used to estimate the information distribution discrimination between two groups of probabilities for the same set of events. Distinguishability is one of the central information-theoretic concepts in science. The distinguishability of language proficiency among L2 learners at different levels can certainly be measured by the information-theoretic method. Relative entropy is an effective algorithm for carrying out this task and it has already been widely applied in various fields of natural science, in the social sciences, and in the humanities [15,16,17,18,19]. In order to illustrate the effectiveness and stability of this new algorithm, we use the traditional frequency-based method and the new algorithm to process the same L2 corpus and compare which algorithm is better.

The current study uses relative entropy to detect lexical and grammatical developments in L2 learners and it is based on a large-scale L2 learners’ corpus (EFCAMDAT2). We will address the following two questions:

(1) How distinct are the differences in language proficiency between L2 learners at a lower level and L2 learners at a higher level as compared to the differences between intermediate-level L2 learners and higher-level learners from the perspective of information gain?
(2) Does the algorithm of relative entropy have advantages over the frequency-based algorithms for lexical and syntactic complexity in detecting development patterns of L2 language proficiency?

## 2. Background

### 2.1. Linguistic Complexity and the Development of Language Proficiency in L2

Linguistic complexity has been extensively studied as an indicator of linguistic performance, development, and proficiency in L2 learners. This means we first need to discuss what linguistic complexity is. A complexity metric quantifies how difficultly a linguistic expression is perceived. However, linguistic complexity has been evaluated using different measures in theoretical linguistics, in applied linguistics and in language cognition. In formal theoretical linguistics, the question of complexity differences among different languages does not arise because the complexity of individual languages is seen as determined by invariant universal mechanisms [20]. In contrast, cross-linguistic complexity differences have long been at the heart of functionalist and usage-based linguistics, particularly in relation to language typology [21,22,23,24,25]. Using information-theoretical measures to detect historical changes in language is also useful [26,27]. 

The research concepts and methods concerning linguistic complexity used in L2 have been reviewed by [28]. Complexity in L2 research has been measured either subjectively through rating scales or, more commonly, through the use of objective quantitative measures [29]. Crucially, L2 research also maintains the distinction between relative complexity (or difficulty) and absolute (or structural) complexity that is used in other disciplines [30,31]. In L2 research, absolute complexity has been associated with the length or size of linguistic units (words, phrases, clauses, sentences, T-units), with the range, variety, richness or diversity of items in a linguistic system or domain, and with properties that refer to the composition and hierarchic organization of linguistic units (e.g., embedding, subordination) [28]. The methods concerning L2 absolute complexity can be summarized using two terms, measures and algorithms, which were all discussed in the Introduction. Measures are linguistic units. In the last 20 years, the studies on lexical/syntactic complexity and development in L2 have played an important role in L2 research, gaining insights of L2 lexical/syntactic acquisition. However, as mentioned in the Introduction section, these advances in the last 20 years are basically finding new and diversified linguistic units to use as measurement in L2. The algorithm is the frequency of these units or the summation (ratio) of two types of frequencies concerning two types of linguistic units. Although new and diversified linguistic units are essential in assessing L2 lexical/syntactic complexity and development, the underlining algorithms also need to be upgraded.

The section of introduction pointed out the main problems with these methods: the algorithm is a little simple and it ignores the meanings and usage of the units. Recent studies have noted the weakness of the measures (linguistic units). In recent studies, it has been proposed to use more fine-grained measures to address such concerns [29]. Yet this weakness has not been completely overcome. 

L2 complexity studies can be applied in order to compare the differences (mostly concerning language proficiency) between L2 learner groups or between L2 learners and L1 learners. These L2 complexity methods usually calculate a score using some given units within a group and they then compare the scores of this group to a number of other groups. These L2 complexity methods assess the differences between these groups concerning language proficiency, learning characters, language difficulty, and language development using the scores so obtained. The reasons for this are that the units measured by different studies are not really the same, and the ‘static’ comparison so discerned is not stable. Additionally, the number of linguistic units/measures used for the assessment is too large (see Appendix C). When many such units/measures are taken to evaluate complexity, this can cause some problems. For example, are these units symmetrical? Which unit is more important? Do too many different units result in a great increase in computational complexity and cost? The studies using these measures/approaches did not explain these difficulties or are simply unaware of these problems. In short, L2 complexity sets out to discern various differences through comparing the scores of given groups. 

Some studies of L2 development have directly discerned the difference between two L2 learner groups. However, these studies, which compared the development of language proficiency between L2 learners or examined differences in language proficiency between L2 learners, focused on “static” data for each L2 level. Because we are concerned with the development of language proficiency, we will concentrate on the difference between the two L2 learner groups directly in a practical manner. Discerning the difference in language proficiency between two L2 learner groups can thus be treated as a “practical and effective” method. 

The other possible area of improvement with respect to previous research on this issue is that many different studies have reported conflicting results concerning the growth of lexical and syntactic complexity in L2 learners. For instance, as the comparison of [1,32] shows, findings reported in different studies can be conflicting even though they were carried out in relatively similar contexts and on learners with a relatively similar proficiency level. However, the challenges that such discrepancies pose lead to more substantive questions. If complexity is achieved by different means at different points (i.e., different proficiency levels) in the developmental paths of L2 learners, then different areas of complexity may be relevant at one given proficiency level but irrelevant or at least less predictive of growth at a different given proficiency level. When these conflicting results concerning developmental patterns in L2 are brought together, the researcher is likely to feel confused and disappointed. The root cause of these conflicting results may be the simplicity of the algorithms, and we have already discussed their drawbacks and possible problems.

A new algorithm could solve these problems by considering the weights of different units that had been previously placed under the same category. However, it is potentially very difficult to assign different weights to fine-grained units. An alternative method that is simpler but operational is to directly measure exactly the same linguistic units in two different groups of L2 learners. Most studies on L2 complexity focused on lexical and syntactic measures, but they overestimated the interface between lexicon and syntax. Few recent studies have examined this area [33,34,35]. This study will include measures at the interface between morphology and syntax. In view of the direct detection of differences and the probability strength, relative entropy should be an operational algorithm for computing the discriminative information distribution through detecting the same set of units in two different groups. 

### 2.2. Relative Entropy

In information theory, entropy [36] quantifies the amount of uncertainty involved in the value of a random variable or the outcome of a random process. “Relative entropy” or the Kullback–Leibler Divergence (KLD, also called “Bayesian surprise”) [37] is derived from entropy. It refers to the number of additional “bits” needed when a non-optimal encoding is used. Relative entropy is actually the expected discrimination information, and it is used to measure the loss and gain of information. Formally, given two probability distributions *p*(*x*) and *q*(*x*) over a discrete random variable *X*, the relative entropy given by *D*(*p*||*q*) is defined as follows:

(1) $$D\left(p∥q\right)=\underset{x\in X}{\sum }p\left(x\right)log\frac{p\left(x\right)}{q\left(x\right)}=\underset{x\in X}{\sum }p\left(x\right)*\left(logp\left(x\right)-logq\left(x\right)\right)$$

*D*(*p*||*q*) (KLD) can also be understood as a measure of the information gained by revising one’s beliefs from the prior probability distribution *q* to the posterior probability distribution *p*. The KLD is therefore used to measure cognitive cost. *P* typically represents the “true” distribution of data or observations, while *q* typically represents ideal data or theoretical data. The order of *p* and *q* in (1) cannot be reversed. However, the KLD is applied to the same underlying set of events. Relative entropy has been widely applied to multiple subjects in science, in social studies, and in the humanities [15,16,17,18,38]. A recent strand of data-driven approaches in the analysis of diachronic change applies KLD measures. For instance, [39] applied the KLD and discovered that there is a correlation between the major intellectual periods in Darwin’s career as identified both by scholarship and his own self-commentary in his reading notes. The KLD was used to analyze the stylistic development of literary works over time in order to discern the patterns of stylistic influence in the evolution of literature [27].

We will illustrate how the KLD is applied to diachronic changes. For instance, the KLD method of a sliding window (e.g., 10 years) can be used to detect changes in units of language. Specifically, the KLD is used to compare preceding (pre-period) and subsequent (post-period) years in the sliding window. We can thus obtain Equation (2) based on Equation (1):

(2) $$\begin{array}{ll} D\left(post∥pre\right)= & \sum_{i}p\left(unit_{i}|post\right)*\left(log_{2}p\left(unit_{i}|post\right)-log_{2}p\left(unit_{i}|pre\right)\right)\\ & \Rightarrow \sum_{i}p\left(unit_{i}|post\right)*log_{2}p\left(unit_{i}|post\right)-\sum_{i}p\left(unit_{i}|post\right)*log_{2}p\left(unit_{i}|pre\right) \end{array}$$

Here, for instance, if the “1800s” are a “pre” period, the “1810s” are a “post” period. The following example is used to illustrate how to compute the KLD. Table 1 is the frequency of the lemma (A lexeme is the set of all forms that have the same meaning, while lemma refers to the particular form that is chosen by convention to represent the lexeme. In English, for example, *run, runs,* and *running* are forms of the same lexeme, but *run* is the lemma.) “take” from 1810 to 1840, which is extracted from the COHA (Corpus of Historical American English, https://www.english-corpora.org/coha/, accessed date: 18 August 2021). When we need to calculate the KLD of “take”, Equation (2) can be specified as follows if “post” and “pre” are the 1820s and the 1810s.

D(1820s||1810s) = ∑*p*(“take”|1820s)*(log_2_*p*(“take”|1820s) − log_2_*p*(“take”|1810s))

= ∑*p*(“take”|1820s)*log_2_*p*(“take”|1820s) − ∑*p*(“take”|1820s)*log_2_*p*(“take”|1810s)

The *lemma* “take” has the five variations, as shown in Table 1. We therefore can obtain the probability for each variation of this lemma in the 1820s:

*p*(“take”|1820s) = 556/1435 = 0.3875,
*p*(“took”|1820s) = 327/1435 = 0.2279,
*p*(“taken”|1820s) = 345/1435 = 0.2404,
*p*(“taking”|1820s) = 144/1435 = 0.1003,
*p*(“takes”|1820s) = 63/1435 = 0.0439.

∑*p*(“take”|1820s)*log_2_*p*(“take”|1820s)

= sum(0.3875*log_2_(0.3875) + 0.2279*log_2_(0.2279) + 0.2404*log_2_(0.2404) + 0.1003*log_2_(0.1003) + 0.0439*log_2_(0.0439)) = −2.041431

Similarly, we can obtain the probability for each variation in the 1810s:

*p*(“take”|1810s) = 728/1415 = 0.5145,
*p*(“took”|1810s) = 168/1415 = 0.1187,
*p*(“taken”|1810s) = 228/1415 = 0.1611,
*p*(“taking”|1810s) = 133/1415 = 0.094,
*p*(“took”|1810s) = 158/1415 = 0.1117.

∑*p*(“take”|1820s) * log_2_*p*(“take”|1810s) = −2.1864

With “take”, D(1820s||1810s) is 0.145 bit. This bit value indicates that the lemma ‘take’ needs an additional 0.145 bit in the 1820s to encode information as compared to the 1810s. This additional bit also suggests that 0.14506-bit more cost was elicited in language users in using this lemma in the 1820s than in the 1810s. The same method is adopted to calculate the KLD of “take” in the 1830s and 1840s, the KLD of the 1830s and 1820s as well as that between the 1840s and 1830s. Therefore, the KLD for the lemma “take” from 1810 to 1840 has the following values: 0.145, 0.0039, 0.0102. These values can be collected to examine how the relative entropy of “take” has changed.

When we talk about distribution information discrimination, the KLD is closely associated with the loss/gain of information. However, when emphasizing language users’ or readers’ cognitive experience, the KLD is related to cognitive cost. Relative entropy has been directly used or adopted to detect cognitive difference or cognitive cost. For instance, tasks that correspond to the encoding of large amounts of information (relative to a model) also have correspondingly higher cognitive costs [40]. The authors of [30] developed a formulation which connects cognitive cost to information cost. However, such information is not available to us because we only have access to the corpus of L2 learners. 

Other information-theoretical metrics have been used to investigate L2 acquisition [35,41]. Previously, information-theoretical measures have been used to evaluate lexical/syntactic complexity [42,43]. For instance, the Kolmogorov complexity algorithm has been used to compute the complexity degree of each L2 learner group [42]. It turned out that more advanced learners use considerably more complex texts than beginner learners. However, Kolmogorov complexity can be used to assess text complexity or utterance complexity rather than grammatical complexity. For instance, the same author may produce different texts with greater or lesser degrees of complexity. Additionally, although the method belongs to information-theoretical metrics, this approach is a “static” approach, and it is hardly capable of capturing the precise differences between L2 learner groups. The authors of [35] employed the scores of the algorithm Pointwise Mutual Information (PMI) to assess collocational complexity of phraseology. Phraseology has been ignored in previous studies of L2 complexity. Although PMI is a measure in information theory that is used to detect associations between two sets of events, it cannot be applied in other types of linguistic phenomena.

## 3. Materials and Methods

### 3.1. Material

The EFCAMDAT2 (*The EF-Cambridge Open Language Database*) currently contains over 83 million words from 1 million assignments written by 174,000 learners across a wide range of levels (The Common European Framework of Reference for Languages, CEFR, the six levels within the CEFR are A1, A2, B1, B2, C1, and C2.). EFCAMDAT2 is naturalistic large-scale L2 corpus compared with the other L2 corpora. This text corpus includes information about learner errors, parts of speech, and grammatical relationships. Researchers can search for language patterns using a range of criteria, including learner nationality and level [44].

The amount of texts from C2 learners (C2 is the highest level in CEFR) according is relatively small as compared to that of the learners at other levels. For this reason, C2 learners will not be considered in the present study. Following [45], the current study took advantage of the information on L2 learner proficiency (A1, A2, B1, B2, and C1) to perform a number of cross-sectional comparisons. The texts in the EFCAMDAT2 came from learners categorized according to 16 proficiency levels that correspond to the six levels of the CEFR: A1 “beginner” (levels 1–3), A2 “elementary” (levels 4–6), B1 “intermediate” (levels 7–9), B2 “upper intermediate” (levels 10–12), C1 “advanced” (levels 13–15), and C2 “proficiency” (level 16). Table 2 summarizes the composition of the 15 different sub-corpora of the written essays section of the EFCAMDAT2 used in our analysis. 

### 3.2. Method

(a) Relative entropy and the discrimination of information distribution.

The algorithm of the KLD can also be used to detect and assess the development of L2 learners’ language proficiency, because the investigation of different levels of L2 learners proceeds in a fashion that is almost the same as the examination of the diachronic changes between several historical sub-corpora that was carried out in previous studies. The differences in language proficiency between L2 learners can be seen as resulting from the differences in the respective amount of time spent learning the language. In this sense, the underlying philosophy behind both investigations is the same. 

This can be applied to examine the differences between L2 learners at the different levels, as shown in the following Equation (3). Here, “*Level_h_*” refers to L2 learners at a higher level, but “*Level_l_*” L2 learners at a lower level.

(3) $$\begin{array}{l} D\left(Level_{h}∥Level_{l}\right)=\sum_{i}p\left(unit_{i}|Level_{h}\right)*\left(log_{2}p\left(unit_{i}|Level_{h}\right)-log_{2}p\left(unit_{i}|Level_{l}\right)\right)\\ \;\;\;\;\;\;\Rightarrow \sum_{i}p\left(unit_{i}|Level_{h}\right)*log_{2}p\left(unit_{i}|Level_{h}\right)-\sum_{i}p\left(unit_{i}|Level_{h}\right)*log_{2}p\left(unit_{i}|Level_{l}\right) \end{array}$$

The KLD can quantify cognitive cost by detecting distinctions in information between the two levels or periods for the same set of events. In Equation (3), a high KLD indicates linguistic novelty in comparison with the lower level. The KLD is also associated with a change in the learner’s reactions upon encountering the unexpected. That means the “*Level_l_*” is the distribution of linguistic phenomena that learners have encountered at a lower level and “*Level_h_*” is the new distribution that learners will encounter at a higher level. More importantly, the algorithm of relative entropy examines the information differences between the same linguistic units encoded by two groups of L2 learners. This avoids the problem that characterized previous studies, namely ignoring the weights of different units and simply placing them under the same category. 

Relative entropy in Equation (2) measures the average amount of additional bits per linguistic unit needed to encode the same linguistic unit distributed according to “L2 learners at a higher level (*Level_h_*)” by using an encoding optimized for “L2 learners at a lower level (*Level_l_*)”. When applied to the comparison of sub-corpora of the EFCAMDAT2, the KLD serves as a strong indication of the degree of difference between two sub-corpora (representing two groups of L2 learners) measured in bits as well as of the linguistic units that are primarily associated with a difference. That is to say, the difference in the KLD indicates that linguistic units need high amounts of additional bits for encoding. We can find the KLD as an indicator of change after sliding over different groups of L2 learners’ lines in the EFCAMDATA2 and by comparing adjacent L2 learners’ groups.

Overall, the KLD detects the discrimination of information distribution for the same set of linguistic units between L2 learners at different levels. In contrast, past methods that focused on the assessment of L2 language proficiency set out to measure one given group of L2 learners within a specific level. This is the biggest difference between the KLD and the past methods. In order to enable cross-verification of the results, we introduced another entropy divergence equation to do the same job. This is the Jensen–Shannon Divergence (JSD) [46]. More details can be seen in Appendix A. The two computational models can be used for the purposes of cross-verification.

(b) Language units (measures).

When we speak of a linguistic “unit”, the term here is confined to lexicon and grammar in their typicality. The “lexicon” has two forms: the first refers to words of all sorts, that is the “token”; the second is the “lemma”, which refers to the canonical form or dictionary form of a set of words. The grammatical forms will be represented by subordinate conjunctions and the POS trigram.

The KLD of lexicon represents the distinction between two groups of L2 learners in information concerning lexical learning. “Token” and “lemma” can ensure that the KLD method really does detect the lexical distinction in the information distribution between the two groups of L2 learners. With respect to the lexicon, all strings containing digits or symbols were removed in order to preserve pure texts for each sub-corpus. After 119 common stop words, one was removed from the lexicon in each sub-corpus, and the lemmas were filtered again by choosing these lexica with a length greater than 2. After the selection, the KLD was calculated using the same vocabulary in each pair of two sub-corpora (two L2 learner groups). As discussed in the Introduction, the algorithm used in previous studies ignored the weights of different linguistic units by placing them under the same category. The relative entropy algorithm computed the same linguistic units for two L2 groups. We used two forms, “token” and “lemma”, to carry out this procedure and finally we obtained the results on KLD tokens and KLD lemmas.

The first grammatical form is the “POS trigram”. The POS trigram refers to a bundle consisting of three words marked by a part of speech. e.g., “wake your dreams” is a trigram and the trigram’s POS is Verb-Pronoun-Noun (abbreviated as “VB-PN-NN”). Being distinct from the bundle of trigram, the POS trigram is an entity consisting of POS labels. According to [47], in practice it is more common to use trigram models in the field of natural language processing because a trigram model depends on the previous two words rather than the previous word. Additionally, according to [48], 3-word lexical bundles have a much higher frequency than 4-word or 5-word lexical bundles. The POS-trigram can play a role in detecting phraseological and grammatical information in texts [34,38,49]. Because of this, we use the trigram POS to present grammar and detect its changes in the sub-corpora of learners at different levels. In this way, the PoS trigram can represent syntactic and phrasal knowledge or information. The raw texts were annotated by *Treetagger* (https://www.cis.uni-muenchen.de/~schmid/tools/TreeTagger/, accessed on 18 August 2021).

In addition to the POS trigram, we used the other measure to detect grammatical differences between L2 learners at different levels. This measure is that of “subordinate conjunction”. Clause subordination has a complex syntactic structure, constituting the potential linguistic complexity, and using it places a higher cognitive demand on language users. Because of these features, clause subordination has been widely investigated in studies of language [50,51,52]. The two features of clause subordination make it very interesting to use to examine people with a limited linguistic competence such as L2 learners [8,53]. The frequency of subordinate conjunctions varies greatly with regard to L2 learners at different levels or with different backgrounds [54,55]. We did not remove any subordinated conjunctions in view of their small number. Each pair of two sub-corpora has the same repository of subordinate conjunctions for calculating the KLD. Note that all of frequencies of the four measures are standard ones, and all the frequencies are based on one million words.

Overall, we have four measures under the categories of lexicon and grammar. The processing of lemmas, tokens, POS-trigrams, and subordinate conjunctions by the KLD will be exactly applied to the JSD, such that the two computational models will take on the same probability distribution as vectors when they are employed to calculate the differences between L2 learners at different levels.

(c) Traditional approaches to lexical/syntactic complexity and stationary time series.

As discussed previously, a number of approaches and tools for lexical and syntactic complexity for L2 based on the algorithm of frequency have been developed and used widely. In order to compare our relative entropy approach with those traditional approaches, we will use the lexical and syntactic complexity approaches [8,9] to process the five sub-corpora of EFCAM2. In this way, we can obtain lexical and grammatical complexities for each corpus representing different L2 levels. However, this is hard to directly compare them with relative entropy. We can obtain the subtraction between two L2 levels and the data on subtraction concerning different L2 levels will be useful in comparing the predictive and explanatory power of the existing methods and indices and the relative entropy approach. Such comparison could provide direct evidence for the assessment of the two approaches. The L2 Syntactic Complexity Analyzer (L2SCA) can analyze the syntactic complexity of written English language for L2 users using 14 different measures. The Lexical Complexity Analyzer (LCA) is able to analyze the lexical complexity of written English language for L2, using 25 measures (see Appendix C).

As discussed above, relative entropy measures information discrimination, so the underlying principle of relative entropy can be applied to examine the differences between L2 learners at the different levels. However, the index of traditional syntactic and lexical complexities cannot be compared to relative entropy. We can apply the principle of difference between L2 learners to calculate the syntactic and lexical gap between two L2 learners. Put it simply, for example, Difference of Syntactic Complexity (A2:A1) = syntactic complexity of A2—syntactic complexity of A1. In this way, we can obtain the difference values among different L2 levels. We will examine these difference values to explore whether these values exhibit certain patterns or not. In other words, we will examine whether the data composed by the difference between L2 levels really follows some patterns. The previous studies simply observed the data on a given L2 level. However, such an examination takes place from a static perspective. A ‘practical and effective’ perspective on L2 development should consider their differences. Overall, given that the data composed by the difference between L2 levels really does follow some patterns, this means the L2 developmental patterns can be recognized explicitly. The approach of difference between two L2 groups is actually one method of time series, which will be specified in the following.

The traditional method for judging the trend in L2 developments has suffered from weaknesses. We can look at language proficiency development in L2 from the perspective of a *time series*. The investigation of different levels of L2 learners proceeds in a fashion that is almost the same as the examination of the diachronic changes that was carried out in previous studies [56,57]. The differences in language proficiency between L2 learners can be seen as resulting from the differences in the respective amount of time spent learning the language. In this sense, the underlying philosophy behind language proficiency between L2 learners concerns the *time series*. Different L2 levels actually constitute a *time series*. That is to say, each L2 level can be observed. In time order, for example, A1 is recorded after L2 learners spend one year, and L2 learners continue to use the other one year to reach A2, and the third year to B1 and so on. Although not all L2 learners use the same amount of time to make continuous progress, many language learning programs for different levels are based on the same time interval, such as language learning in formal schools based on a semester system. As another example, much of the data collection for language proficiency in longitudinal studies is based on the same time interval [2,7]. In this sense, the data on different L2 levels is basically a sequence taken at successive equally spaced points (e.g., one year) in time. Thus, it is a sequence of discrete-time data, that is, *time series*. According to the time series statistics, a direct comparison between the values is somewhat unreliable. 

Although many studies concerning L2 proficiency development have treated differences between L2 levels as time differences, the statistics of *time series* has seldom been employed to process the data on L2 difference. For this reason, it is promising to use the *time series* perspective to examine the data yielded by traditional methods. Borrowing the principle of relative entropy and the stationary and differencing method in *time series* [58,59], we used the “first difference” to examine the data on lexical/syntactic complexity of L2 by traditional algorithms. Differencing is performed by subtracting the previous observation from the current observation. It can be simply described as “Difference(t) = observation(t) − observation(t − 1)”. Here, t refers to time (time order). When the data on Difference (t, t + 1, t + 2, …, t + n) does not show any increase or decrease, this indicates that the original data are stationary. This means that there is no pattern depending on the time at which the series are observed.

## 4. Results

### 4.1. The Results from the KLD

The results of the KLD of lexicon and grammar regarding five sub-corpora of the EFCAMDAT2 can be seen in the following three tables. They are displayed from different perspectives.

Table 3 presents the KLD results which examine relative entropy from the perspective of L2 at a lower level. Note that in Table 3, the upward arrow represents an upward trend in the data of this group, while the downward arrow represents a downward trend in the data in that group. The upward arrow and the downward arrow used in the other tables have the same function. As for a group of relative entropy data, we have used linear regression model to test whether the values in this group have an increase trend or not. “*Coef*” is the abbreviation of coefficient, representing the coefficient of this linear model, and *p*-value helps judge whether this linear model is significant or not.)

Table 3 shows that the KLD between a given lower level (e.g., A1, A2, B1) and a higher level becomes larger when L2 learners’ level increases. However, the KLD of token starting from A2 level and that of token and lemma starting from the B1 level is an exception. We used linear regression to examine whether the data in each group has a significant increase or decrease. When *coef* is positive, it means that these data have an increase and a negative *coef* indicates a decrease. When the *p* value is greater than 0.05, this suggests that such an increase or decrease may be not significant. The linear regression model is just used for a general observation of the direction of the data, so that it can be compared with the results yielded by traditional methods. For example, after one group data under “POS-trigram”, “0.37, 0.58, 0.78, 1”, is examined by a linear regression, we find that the coefficient is 0.21 and *p*-value is smaller than 0.001. This indicates that an increase in this group is significant, so there is an upward arrow. Because there are few numbers within a group data, the regression models are only auxiliary (the *p*-value is only a reference and not decisive). It is actually easy to tell whether the numbers within a group are increasing or decreasing. These are applied in Table 3, Table 4 and Table 5, as well as Table A1, Table A2 and Table A3 of Appendix B. From the perspective of cognitive cost, the KLD tends to increase over different groups of L2 learners if the starting point is A1. Specifically, the upward trend is 75% (9/12) of all measurement units. This could suggest that L2 learners at a lower level find it more difficult to process these language units than L2 learners at a higher level. Overall, the KLD between L2 learners at a higher level and L2 learners at a lower level is much larger than that between L2 learners at a lower level and L2 learners at an intermediate level. This indicates that L2 learners at a lower level need larger amounts of additional bits for encoding these linguistic units in comparison to L2 learners at a higher level. We will now look more closely at the KLD between L2 learners from the perspective of the higher levels.

As Table 4 shows, the KLD between a higher level and a lower level is much larger than the KLD between a lower level and an intermediate level. The KLD drops when the level moves from the lower to higher. This indicates that the gap between the level of the learners’ degree of language proficiency is proportional to their KLD. Specifically, the downward trend is 100% (12/12) of all measurement units. This could suggest that the difference of language proficiency between L2 learners at a higher level and L2 learners at an intermediate level is smaller than that between learners at the intermediate level and learners at the lower level. It also indicates that L2 learners at a higher level need less additional bits for encoding these linguistic units in comparison to L2 learners at a lower level. This also suggests that L2 learners at a higher level do not have a greater degree of cognitive cost in processing these linguistic units than L2 learners at a lower level. The following examines the KLD of L2 learners at adjacent levels.

Table 5 shows that when language levels become higher, their KLDs tend to become smaller. This could indicate that learners at intermediate levels have fewer difficulties in learning lexicon and grammar in the course of improving their language proficiency. By contrast, learners at the lower level may need to make more effort in improving their language proficiency. Specifically, the upward trend is 75% (3/4) of all measurement units. The data on the KLD in the three tables are consistent in supporting the thesis that the KLD between higher levels is much smaller than that between lower levels or that between a lower level and an intermediate level. 

**Figure 1:**
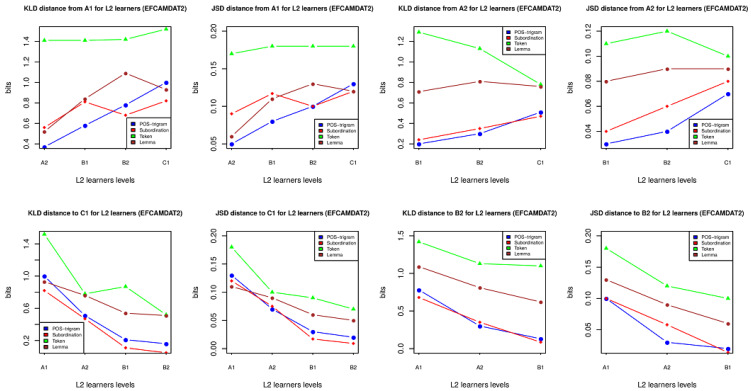
Relative entropy among L2 learners at cross-proficiency different levels (EFCAMDAT2). Note that JSD results are also visualized in this figure.

We represented some of the data from the above tables in Figure 1 so as to present the changes of the KLD between L2 learners at various levels more clearly. In each plot of Figure 1, *x-axis* represents L2 proficiency level and *y-axis* stands for relative entropy value (bits). Given that the curve shows an upward or downward trend, it indicates that relative entropy can detect patterns of L2 proficiency growth. However, when a curve looks irregular, it means that relative entropy fails. Alternatively, when a curve has a different trend compared with the other curves in a plot, it suggests that this curve also fails to capture patterns of L2 development. Additionally, a flat curve indicates no clear growth. For example, in the right top two plots, there is one exception where the token has a clearly opposing trend to the other measures. The four plots on the top clearly show that relative entropy increases in the majority of cases given we view from A1 to a higher level. By contrast, there is a clear decrease of relative entropy in the bottom four plots in Figure 1 given we view from C1 to a lower level. As discussed above, the top four plots are consistent with the bottom four plots, that is, relative entropy is effective to detect the linear change of language proficiency among L2 at different levels. Figure 1 and the data from the tables above actually answer the first research question posed at the end of the Introduction. 

To save space, we have placed the results of the JSD in Appendix A. The results from the JSD are almost the same as those of the KLD. Overall, the JSD data are consistent with those of the KLD. 

### 4.2. The Results from Syntactic and Lexical Complexity

**Figure 2:**
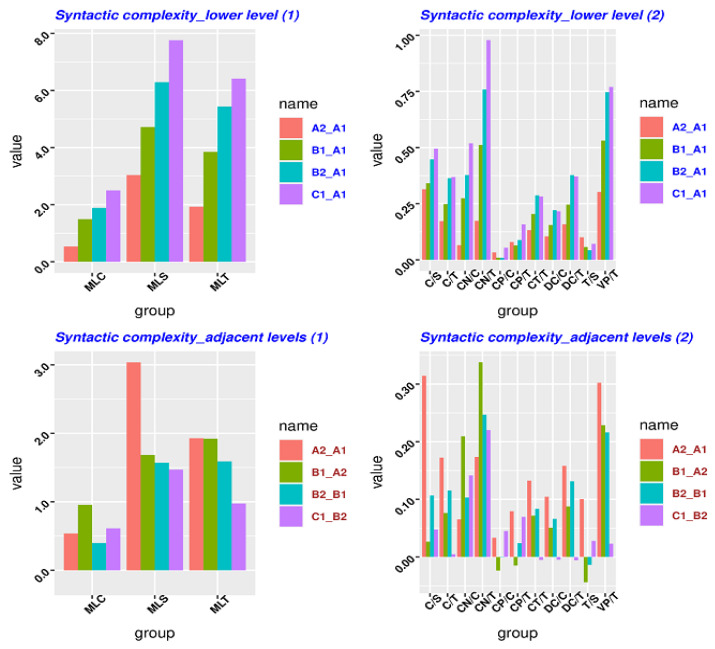
The difference of syntactic complexity between different L2 levels. Here, each L2 level (A1, A2, B1, B2, C1) can be treated as *time order* (date). Here, *x-axis* is syntactic complexity measures/metrics, and *y-axis* is the difference of those complexity metrics across proficiency levels (discussed in the section of Methods). The left top plot shows that MLC, MLS, and MLT have a gradual increase, that is, B1_A1 is higher than A2_A1, and B2_B1 is higher than B1_A1, and C1_B2 is higher than CB2_B1. When a metric shows a regular increase, it indicates that this measure can detect patterns of L2 proficiency development. By contrast, in the right top plot, such a regular increase can only be found in 4 of the 12 metrics. In the bottom two plots, none of metrics shows a regular increase. Irregular changes suggest that these metrics cannot capture the patterns of L2 proficiency development.

We used L2SCA and LCA to process the five sub-corpora. Due to the limit of the length, the original data are shown in Appendix C. Table A4 of Appendix C shows that the original data on syntactic complexity (measured by different metrics) for each L2 level and the gap between a given lower level (i.e., A1) and a higher level increases when L2 learners’ level of linguistic proficiency rises. Table A5 of Appendix C shows that the original data on lexical complexity (measured by different metrics) for each L2 level and the gap between a given lower level (i.e., A1) and a higher level increases when L2 learners’ level of linguistic proficiency rises. As discussed in the Methods section, it is hard to compare relative entropy with the data on syntactic or lexical complexity for each level. According to the *time series* perspective, each L2 level is treated as a date. The gap between two L2 levels is the difference between the two dates, which has been discussed in the section of Methods. The other benefit is that the gap between two levels represented by syntactic or lexical complexity can be used to compare relative entropy because they share the same concept. In order to make a better observation, we represented in Figure 2 the data contained in Table A4 and Table A5 of Appendix C. 

Figure 2 is composed of four panels. The top two panels represent the syntactic complexity gap between a given lower level (i.e., A1) and a higher level. All syntactic complexity metrics should be represented in one plot. However, the values in the three metrics (i.e., MCL, MLS, MLT) are somewhat larger than the others, so these three are plotted in a different graph to allow the data to be easily seen. The two panels at the bottom represent the syntactic complexity gap between the two adjacent levels (e.g., A1–A2, A2–B1). For this reason, we have represented syntactic complexity from the perspective of adjacent levels in the same way. 

The top panel has deviant groups: CP/C, CP/T, DC/C, DC/T, T/S (5/14). It means that 36% of syntactic measures do not show patterns from the perspective of the low level (A1). The bottom panel has deviant groups: C/S, C/T. CN/C, CN/T, CP/C, CP/T, CT/T, DC/C, DC/T, ML/C, T/S (11/14). This indicates that almost 80% of syntactic measures do not show patterns from the perspective of the adjacent level. None of the metrics exhibit patterns from the perspective of the higher level (C1), as shown in Table A4 of Appendix C. In comparison with Table 4 and Table 5, in Table A2 and Table A3 of Appendix B (KLD and JSD) the measures of traditional syntactic complexity do not perform better than relative entropy because the syntactic measures of relative entropy form more regular patterns than those of traditional syntactic complexity. 

**Figure 3:**
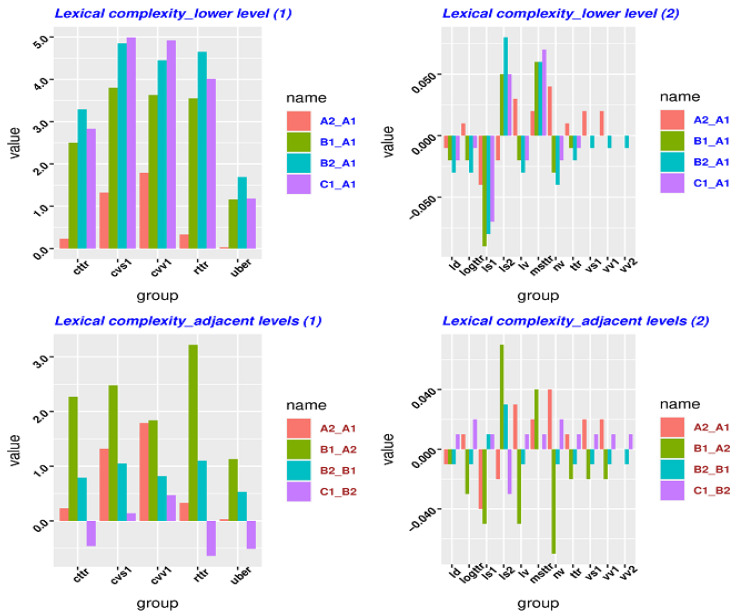
The difference of lexical complexity between different L2 levels.

Figure 3 is also composed of four panels. The top two panels represent the lexical complexity gap between a given lower level (i.e., A1) and a higher level. All lexical complexity metrics should be represented in one plot. However, the values in the three metrics (i.e., cttr, cvs1, cvv1, rttr, uber) are somewhat larger than the others, so the three are plotted in a different graph so as to allow easy observation. The bottom two panels represent the lexical complexity gap between the two adjacent levels (e.g., A1–A2, A2–B1). For this reason, we have represented the lexical complexity from the perspective of adjacent levels in the same way. 

The top panel has deviant groups: uber, ld, logttr, ls1, ls2, lv, msttr, nv, ttr, vs1, vs1, vv1, vv2 (13/16). This means that 81% of metrics do not show patterns from the perspective of the low level (A1). The bottom panel has deviant groups: cttr, rttr, uber, ld, logttr, ls1, ls2, lv, msttr, nv, ttr, vs1, vs1, vv1, vv2(15/16). It indicates that 94% of metrics do not show patterns from the perspective of the adjacent levels. The perspective of higher level (C1) reveals that no metrics exhibit any patterns, as shown in Table A5 of Appendix C. In comparison with Table 4 and Table 5, in Table A2 and Table A3 of Appendix B (KLD and JSD) the measures of traditional lexical complexity do not perform better than relative entropy. Although the lexical measures of relative entropy do not perform better than the syntactic measures of relative entropy, these lexical measures still exhibit more regular patterns than the traditional measures.

More importantly, the data in Figure 2 and Figure 3 can be taken as a *Difference type* from the perspective of *time series* because the *Difference data* comes out by subtracting from the data in one state with the data from the other state (i.e., “Difference(t) = observation(t) − observation(t − 1)”, t = time), as discussed in Section 3.2 (c). It turns out that the Difference data in Figure 2 and Figure 3 does not show any increase or decrease patterns. It indicates that the original data yielded by the traditional lexical/syntactic measures (as seen in Table A4 and Table A5 of Appendix C) is stationary. In other words, it does not matter when the original data is observed because the original data should look much the same at any point in time. In this sense, the data on lexical/syntactic complexity measured by the traditional methods at different L2 levels does not exhibit an increase or decrease trend from the perspective of *time series*. Instead, relative entropy is helpful in showing more regular patterns of language proficiency among the different L2 levels. Specifically, 75%, 100%, and 75% of metrics show patterns using KLD respectively, shown in Table 3, Table 4 and Table 5; 75%, 100%, and 100% of metrics using JSD respectively show patterns. By contrast, as analyzed above, under the framework of traditional methods, 20% and none of syntactic metrics show patterns, and 19% and 6% of lexical metrics show patterns. Overall, the algorithm of relative entropy is much more effective and stable in detecting language proficiency development in L2 than the traditional frequency-based methods.

## 5. Discussion

The following section discusses our findings and compares them with the findings reported in previous related studies. As discussed above, the strength of relative entropy lies in detecting the differences. We explain how our findings are to be interpreted and how they relate to the findings in the previous studies from three points of view. This will address the second research question.

### 5.1. Conflicting Results from Different Studies

In Section 2.1, we mentioned the conflicting results reported by various studies of L2 development. For instance, [53] proposed that global complexity (for example, the mean length of T-unit) can most likely capture overall changes in complexity in any data. They further predicted that subordination, for example, as measured by the mean number of clauses per T-unit, is the preferred means of understanding linguistic complexity used at intermediate levels. At the most advanced levels of proficiency, subordination would cease to be predictive, and they instead expect that phrasal elaboration would be the main area of growth. However, when we closely consider the syntactic areas of complexity, the findings across different studies can look disconcerting. For instance, [1] found the opposite pattern: at the upper-intermediate level, their sample showed phrasal elaboration changes, but subordination remained unchanged. Both phrasal and clausal complexity grow together with regard to lower-proficiency L2 writers. Such conflicting results can be frustrating for researchers [2]. 

As discussed in the Introduction and Background sections, the main reason for these conflicting growth assessments is that all the measures used therein were based on traditional measures, which only compute the scores for each L2 learner group. These traditional measures are greatly influenced by the corpus size and the diversity of texts. When different types of static data are collected, comparison reveals that their results conflict. For instance, using again the example of ‘subordination’ that was mentioned in the Introduction, L2 learners at the A1 level use a “that-clause” 10 times, while L2 learners at the B2 level use various “time, contrast, reason-effect” adverbial clauses 10 times. The algorithm based on the summation or ratio of frequencies tends to treat the two cases as the same in their use of subordination. The result of this is that L2 learners at the two levels seem to remain stable with regard to their use of ‘subordination’. Suppose that L2 learners at the B2 level use various “time, contrast, reason-effect” adverbial clauses 9 (lower than 10) times. It seems on this approach that L2 learners at the B2 level use less complex subordinate structures than learners at the A1 level. However, the real situation is the opposite to that given in the results from the algorithm of summation or the ratio of frequencies. The same mistake occurs when the algorithm for measuring lexical or phrasal complexity is applied because it does not examine the exact linguistic units when using these measures and thus cannot compute the diversity of given linguistic units. Clearly, these conflicting results do not necessarily challenge the assumption that as L2 development progresses, learners use more elaborate, complex, and diverse language as regards lexicon and grammar. 

### 5.2. The Developmental Patterns of Language Proficiency in L2 Learners

The findings reported in Section 4.2 allow us to interpret these conflicting reports to some degree. Clearly, linear growth or fluctuating development was reported by various studies. However, the summation of frequency or the ratio of two types of frequencies means they cannot really be representative of L2 complexity. Consequently, the change in the summation scores or ratios cannot really represent the L2 development with respect to language proficiency.

As a matter of fact, it is more practical to describe the difference between the two groups of L2 learners rather than applying an indirect method. It is possible that L2 learners at a higher level could produce more complex structures with respect to some linguistic phenomena. However, L2 learners at a higher level could provide some simpler structures with respect to these linguistic phenomena. For instance, while beginning and intermediate L2 learners may prefer complexity through coordination and subordination, phrasal complexity may be favored at more advanced levels of L2 proficiency [2]. This means that L2 advanced learners could increase the degree of complexity in subordination. This is confirmed by [60] who found that the learners produce longer, more complex phrases (more modifiers per noun phrase) at the end of a course of study, but not more or even fewer, subordinated clauses (i.e., fewer verb phrases; there is no significant change in number of subject relative clauses or ‘‘that’’ verb complements). These findings are not consistent with a linear growth or development in complexity. However, the complexity measured in the algorithm of frequency could have changed to detect the diversity in this usage of subordination in L2 learners at different levels, as was explained in the Introduction. It is highly likely that the information distribution concerning subordination might show greater differences between L2 learners at a lower level and L2 learners at a higher level than L2 learners of an intermediate level and a higher level. 

As shown in Table A4 and Table A5 of Appendix C as well as in Figure 2 and Figure 3, the traditional syntactic and lexical approaches do not really seem to capture the developmental patterns of language proficiency in L2 learners so well. Different metrics even show conflicting trends. In many cases, these metrics demonstrate irregular patterns. The *Different data* in *time series* also demonstrates that the data yielded by the traditional lexical and syntactic measures is one stationary type. In another word, these data can show few regular patterns in L2 language proficiency development. 

When these metrics exhibit irregular patterns or even conflicting patterns, the possibility that the underlying algorithm (frequency, or ratio) behind these traditional measures is not really stable or effective, which has seldom been considered previously. Our data, which was yielded by relative entropy, reveals that the information distribution discrimination regarding lexical and grammatical differences continues to increase from L2 learners at a lower level to those at a higher level. This finding is consistent with the assumption that as language proficiency develops, L2 learners will acquire a more elaborate, complex, and diverse command of the language. In addition to this, the developmental pattern we found is consistent with the differences in language proficiency among these L2 learners. This means that our method can detect the development of language proficiency among L2 learners at different levels more robustly and stably. We have thus addressed the second research question. 

Both the methods we developed and the findings could provide us inspiration for second language acquisition, language assessment, and language teaching programs. Firstly, we have developed a practical and effective method to detect the language proficiency development of L2 learners. Secondly, our findings suggest that L2 proficiency development may be towards a more complex and diverse use of language, which is consistent with our intuition. In another word, L2 learners may acquire a second language linearly. Thirdly, our finding can greatly help gain insight in L2 acquisition and language teaching programming, which will be discussed in greater detail. For example, at certain stages, the focus of instruction will change. Vocabulary instruction may be less of a focus when teaching L2 learners at a higher level, and more of a focus when teaching complex sentence structures. For those L2 learners, the focus may be on textual structure rather than sentence structure. However, the linear development in L2 lexicon and syntax may not be influenced by the fact that the teaching emphasis could have been put elsewhere (i.e., textual structure). The reason for this is that textual focus still helps L2 learners to improve their lexical and syntactic capabilities. However, some past studies thought that their syntactic capabilities would stop increasing when L2 learners are at the intermediate level. Accordingly, the curriculum should make changes to emphasize teaching sentence structure again for L2 learners at the intermediate level. However, our finding may suggest that an overemphasis on curriculum changes to cater to those past research findings based on the traditional methods may not achieve the results they are looking for. Further, the method of relative entropy can not only be applied in detecting English L2 proficiency development, but also in detecting L2 proficiency development in the other languages. Moreover, relative entropy can be applied in lexical and grammatical change patterns in across-genre and across-language studies. Although the past studies have applied relative entropy in across-genre and across-language studies, they mostly used lemma as their measurement units. The linguistic units we have employed (i.e., POS-trigam, conjunction) can be applied in these fields help reveal more findings. 

### 5.3. Consistency with the Other Measures

Relative entropy thus has unique strengths in comparison with the previous methods when it comes to measuring the development of language proficiency in L2 learners. The present study shows that the relative entropy of lexicon is largely consistent with the relative entropy of grammar. In past studies, results that were calculated by the frequency algorithm based on fine-grained linguistic units were found to be inconsistent with those yielded by non-fine-grained linguistic units. We also discussed another type of inconsistency in previous studies, namely that between syntactic measures and lexical ones. However, such inconsistencies have not been interpreted satisfactorily. These inconsistencies indicate that these measures and their underlying algorithm are not necessarily a good predictor of L2 learners’ language proficiency and its development. 

Many studies using the traditional algorithm actually make a post-hoc analysis. That is to say, after these studies have obtained their results, these are then compared with the language proficiency levels of L2 learners in an attempt to find out something that explains the connection between their data and these levels of language proficiency. By contrast, the change of distinctions in relative entropy is consistent with the discrepancy between the levels representing L2 learners’ language proficiency. Relative entropy can thus be treated as a measure for predicting L2 learners’ language proficiency. More importantly, all measures (metrics) in the traditional methods (see Appendix C) must be interpreted individually. That is to say, the summation or ratio of frequency cannot provide a consistent interpretation for these measures. For instance, the three measures, C/T (# of clauses/# of T-unit), CT/T (# of complex T-units/# of T-units), DC/C (# of dependent clauses/# of clauses), have to be interpreted differently. When these units of measurement have different interpretations, various factors (gender, prompt-task, nationality, etc.) will be incorporated to explain these differences and conflicting results, and all seem to have plausibility.

The other advantage of relative entropy is that it can be used to interpret cognitive distinctions among L2 learners. Relative entropy can be treated as a simpler version of cognitive cost although the formulation for evaluating cognitive cost in tasks is a bit more complex than the KLD. Cognitive cost/effort can be interpreted as a subjective feeling of exhaustion experienced when performing a cognitive task and its associated task-avoidance. The cognitive costs associated with different classes of tasks are known to be subjectively demanding. It has been showed that this informational perspective can provide a unitary perspective on various experimental findings. Furthermore, we have discussed how information costs could be translated into cognitive effort (i.e., the subjective feeling associated with performing costly tasks). According to Table 3, Table 4 and Table 5, the cognitive cost on the part of L2 learners at a lower level and L2 learners at an intermediate level is much larger than that between L2 learners at an intermediate level and L2 learners at a higher level. This could reflect a decrease in cognitive cost that takes place when L2 learners acquire a greater language proficiency. It could be that L2 learners at a lower level make greater cognitive efforts in improving their language proficiency. This finding may indicate that a L2 learner at a lower level may need more instructions and practices to improve their language capabilities. By contrast, L2 learners at a higher level will feel it is easier to improve their language proficiency. 

## 6. Conclusions

The current study used a novel ‘practical and effective’ algorithm derived from information-theoretic metrics to discern the development of L2 learners’ acquisition of language proficiency and it was based on a large-scale L2 writing corpus. It turned out that the relative entropy of lexical and grammatical differences continues to increase from L2 learners at a lower level to those at a higher level. This finding is consistent with the assumption that L2 learners acquire a more complex and diverse language as they progress. It is also consistent with the different levels of L2 language proficiency. Another key finding of this study is that relative entropy is a better predictor of language proficiency than the algorithm based on frequency summation or ratio. The *Different* data in *time series* demonstrates that the data yielded by the traditional lexical and syntactic measures can show few regular patterns in L2 language proficiency development. This means that the traditional approach seems not to yield robust, and stable results in detecting developmental patterns in L2. By contrast, the algorithm of relative entropy reveals that the information distribution discrimination regarding lexical and grammatical differences continues to increase from L2 learners at a lower level to those at a higher level. Our findings also offer insights into the cognitive aspect of the development of L2 language proficiency, namely that L2 learners at a lower level have to make greater cognitive efforts to improve their language proficiency. All this indicates that as compared to the algorithm of frequency summation and ratio, the ‘practical and effective’ algorithm that uses relative entropy could be more effective and stable when it comes to detecting differences of linguistic complexity between L2 learners than the frequency-based algorithm that was previously used. 

Overall, as far as it seems, at least we have found a possible correct direction. In spite of this, there is still much to be done to improve the current algorithm, for example, by considering more units of measurement (discourse connectives, stance words, etc.). Moreover, we will consider the (random) factors available in L2 corpus (gender, age, nationality, text length, etc.). It would be better to control for these factors to further explore how they take effect on relative entropy using generalized mixed-effects statistical models. Such explorations could make relative entropy detect L2 proficiency development in a more reliable and solid manner.

## Figures and Tables

**Table 1:** Frequencies of “take” and its tokens from 1810s to 1840s.

Word	1810s	1820s	1830s	1840s
Take	728	556	665	529
Took	168	327	351	333
Taken	228	345	344	324
Taking	133	144	164	165
Takes	158	63	76	86
**Total**	1415	1435	1600	1437

**Table 2:** Composition of the five sub-corpora of the essays section of the EFCAMDAT2 by language proficiency level.

L2 Learners’ Proficiency Levels	Texts	Learners	Tokens	Lemmas
A1	625,985	103,742	28.8 M	27,065
A2	307,996	52,734	24 M	32,051
B1	168,361	32,852	18.4 M	26,276
B2	61,329	13,951	9.3 M	21,312
C1	14,698	2839	2.8 M	16,464

**Table 3:** The KLD results from the perspective of L2 at a lower level.

Cross-Proficiency Levels of L2		KLD of Grammar				KLD of Lexicon			
		POS-Trigram		Sub-Conj.		Token		Lemma	
A1→A2	A1→(A2, B1, B2, C1)	0.37	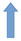 (coef = 0.21, *p* < 0.001)	0.56	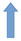 (coef = 0.07, *p* = 0.31)	1.41	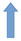 (coef = 0.03, *p* = 0.18)	0.52	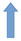 (coef = 0.15, *p* = 0.2)
A1→B1		0.58		0.81		1.41		0.84	
A1→B2		0.78		0.68		1.42		1.09	
A1→C1		1.0		0.82		1.52		0.93	
A2→B1	A2→(B1, B2, C1)	0.2	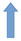 (coef = 0.16, *p* = 0.13)	0.24	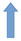 (coef = 0.12, *p* = 0.016)	1.29	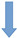 (coef = −0.25, *p* = 0.13)	0.71	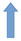 (coef = 0.025, *p* = 0.66)
A2→B2		0.3		0.35		1.13		0.81	
A2→C1		0.51		0.47		0.78		0.76	
B1→B2	B1→(B2, C1)	0.13		0.085		1.1		0.62	
B1→C1		0.21		0.11		0.87		0.54	
B2→C1	B2→(C1)	0.16		0.052		0.52		0.51	

**Table 4:** The KLD results from the perspective of L2 at a higher level.

Cross-Proficiency Levels of L2		KLD of Grammar				KLD of Lexicon			
		POS-Trigram		Sub-Conj.		Token		Lemma	
A1→C1	(A1, A2, B1, B2)→C1	1.0	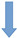 (coef = −0.28, *p* = 0.056)	0.82	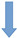 (coef = −0.27, *p* = 0.034)	1.52	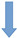 (coef = −0.29, *p* = 0.12)	0.93	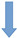 (coef = −0.15, *p* = 0.034)
A2→C1		0.51		0.47		0.78		0.76	
B1→C1		0.21		0.11		0.87		0.54	
B2→C1		0.16		0.05		0.52		0.51	
A1→B2	(A1, A2, B1)→B2	0.78	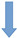 (coef = −0.33, *p* = 0.171)	0.68	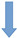 (coef = −0.3, *p* = 0.04)	1.42	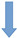 (coef = −0.16, *p* = 0.28)	1.09	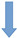 (coef = −0.24, *p* = 0.07)
A2→B2		0.3		0.35		1.13		0.81	
B1→B2		0.13		0.085		1.1		0.62	
A1→B1	(A1, A2)→B1	0.58	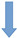	0.81	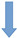	1.41	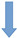	0.84	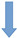
A2→B1		0.2		0.24		1.28		0.71	
A1→A2	(A1)→A2	0.37		0.56		1.41		0.52	

**Table 5:** The KLD results from the perspective of adjacent L2 levels.

Cross-Proficiency Levels of L2		KLD of Grammar				KLD of Lexicon			
		POS-Trigram		Sub-Conj.		Token		Lemma	
A1→A2	adjacent levels	0.37	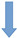 (coef = −0.07, *p* = 0.16)	0.56	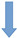 (coef = −0.16, *p* = 0.086)	1.41	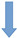 (coef = −0.28, *p* = 0.063)	0.52	
A2→B1		0.2		0.24		1.28		0.71	
B1→B2		0.13		0.085		1.1		0.62	
B2→C1		0.16		0.081		0.52		0.51	

## Appendix A. JSD Algorithm

The usefulness of KL is simply that it is the fundamental building block of the JS divergences. An alternate approach is the JS divergence, which is another method of measuring the similarity between two probability distributions. It is a symmetric and smoothed version of the KL divergence, and it can be used as a distance metric. The JSD has also been widely applied in various fields [19,61,62]. The JS divergence is symmetrical in two distributions and the weights are each ½. Defining the quantity M = (P + Q)*(0.5), we can write the JS divergence as:

JS (p||q)= 1/2*KLD(p||(p + q)/2) + 1/2*KLD(q||(p + q)/2), (A1)

JS(Level_h_||Level_l_)= 1/2*KLD(Level_h_||(Level_h_ + Level_l_)/2) + 1/2KLD(Level_l_||(Level_h_ + Level_l_)/2). (A2)

In Equation (A1), the KLD is the KL divergences in the above equations. Similarly, we can obtain the JSD for the lemma “take” in Table 1: JSD = (1820s||1810s) = 0.0249; JSD(1830s||1820s) = 0.0007; JSD(1840s||1830s) = 0.0014.

This subsection presents the results calculated by the JSD. As discussed in the Method section, the strength of the JSD is that it has a symmetrical and smoothed function in comparison to the KLD. More importantly, the results from the JSD can be used to cross-verify the KLD in order to ensure the validity and consistency of its results. Similar to the KLD, the JSD is able to detect the discrimination of information distribution between L2 learners at different levels. KLD and JSD can cross-validate with each other.

## Appendix B. The Results from the JSD

This subsection presents the results calculated by the JSD. As discussed in the Method section, the strength of the JSD is that it has a symmetrical and smoothed function in comparison to the KLD. More importantly, the results from the JSD can be used to cross-verify the KLD in order to ensure the validity and consistency of its results.

Table A1 shows that the JSD between a given lower level (e.g., A1, A2, B1) and a higher level increases when L2 learners’ level of linguistic proficiency rises. However, there is an exception in the JSD of token starting from A2 level and that of token and lemma starting from B1 level. There the JSD values remain stable. Specifically, the upward trend is 75% (9/12) of all measurement units. Overall, this could suggest that the JSD between L2 learners at a higher level and L2 learners at a lower level is much larger than that between L2 learners at a lower level and L2 learners at an intermediate level. This indicates that L2 learners at a lower level need a greater amount of additional bits for encoding these linguistic units in comparison with L2 learners at a higher level. It also suggests that L2 learners at a lower level have a greater degree of cognitive cost in processing these linguistic units than L2 learners at a higher level. In short, the JSD data in Table A1 is consistent with the KLD data in Table 3.

**Table A1:** JSD results from the perspective of L2 at a lower level.

Cross-Proficiency Levels of L2		JSD of Grammar				JSD of Lexicon			
		POS-Trigram		Sub-Conj.		Token		Lemma	
A1→A2	A1→(A2, B1, B2, C1)	0.05	(coef = 0.03, *p* = 0.003)	0.09	(coef = 0.007, *p* = 0.34)	0.17	(coef = 0.003, *p* = 0.23)	0.06	(coef = 0.02, *p* = 0.17)
A1→B1		0.08		0.117		0.18		0.11	
A1→B2		0.1		0.1		0.18		0.13	
A1→C1		0.13		0.12		0.18		0.12	
A2→B1	A2→(B1, B2, C1)	0.03	(coef = 0.2, *p* = 0.18)	0.04	(coef = 0.02, *p* < 0.001)	0.11		0.08	(coef = 0.005, *p* = 0.33)
A2→B2		0.04		0.06		0.12		0.09	
A2→C1		0.07		0.08		0.1		0.09	
B1→B2	B1→(B2, C1)	0.02		0.014		0.1		0.06	
B1→C1		0.03		0.017		0.09		0.06	
B2→C1	B2→(C1)	0.02		0.009		0.07		0.05	

As Table A2 shows, the JSD between a higher level and a lower level is much larger than that between a lower level and the intermediate levels. The JSD drops when the level moves from the lower to the higher. It indicates that the distinction between different groups is proportional to their JSD. Specifically, the downward trend is 100% (12/12) of all measurement units. This could mean that the difference in language proficiency between L2 learners at a higher level and learners at an intermediate level is smaller than that between learners at the intermediate level and learners at the lower level. The JSD data in Table A2 is completely consistent with the KLD data in Table 4. The following examines the JSD of adjacent levels.

**Table A2:** JSD results from the perspective of L2 at a higher level.

Cross-Proficiency Levels of L2		JSD of Grammar				JSD of Lexicon			
		POS-Trigram		Sub-Conj.		Token		Lemma	
A1→C1	(A1, A2, B1, B2)→C1	0.13	(coef = −0.04, *p* = 0.043)	0.12	(coef = −0.04, *p* = 0.034)	0.18	(coef = −0.034, *p* = 0.09)	0.11	(coef = −0.02, *p* = 0.015)
A2→C1		0.07		0.075		0.1		0.09	
B1→C1		0.03		0.017		0.09		0.06	
B2→C1		0.02		0.009		0.07		0.05	
A1→B2	(A1, A2, B1)→B2	0.1	(coef = −0.04, *p* = 0.26)	0.1	(coef = −0.043, *p* = 0.008)	0.18	(coef = −0.04, *p* = 0.18)	0.13	(coef = −0.035, *p* = 0.05)
A2→B2		0.03		0.058		0.12		0.09	
B1→B2		0.02		0.014		0.1		0.06	
A1→B1	(A1, A2)→B1	0.08		0.12		0.18		0.1	
A2→B1		0.03		0.04		0.11		0.08	
A1→A2	(A1)→A2	0.05		0.09		0.17		0.06	

Table A3 shows that when levels become higher, their JSDs tend to become smaller. It could be that learners at intermediate levels have fewer difficulties in learning lexicon and syntax in the course of improving their language proficiency. By contrast, learners at the lower level may need to make more effort in improving their language proficiency. Specifically, the upward trend is 100% (4/4) of all measurement units. The data on the JSD in the three tables are consistent in supporting the thesis that the JSD between higher levels is much smaller than that between lower levels or between a lower level and an intermediate level. Overall, the JSD data are completely consistent with that of the KLD. The visualization of JSD results is presented in Figure 1 of the body text.

**Table A3:** JSD results from the perspective of adjacent L2 levels.

Cross-Proficiency Levels of L2		JSD of Grammar				JSD of Lexicon			
		POS-Trigram		Sub-Conj.		Token		Lemma	
A1→A2	adjacent levels	0.05	(coef = −0.01, *p* = 0.087)	0.09	(coef = −0.027, *p* = 0.06)	0.17	(coef = −0.03, *p* = 0.046)	0.06	(coef = −0.005, *p* = 0.48)
A2→B1		0.03		0.04		0.11		0.08	
B1→B2		0.02		0.015		0.1		0.06	
B2→C1		0.02		0.009		0.07		0.05	

## Appendix C. The Data on Syntactic and Lexical Complexity

These syntactic metrics in L2SCA are included as follows: Length of production unit: MLC (# of words/# of clauses), MLS (# of words/# of sentences), MLT (# of words/# of T-units). Amount of subordination: C/T (# of clauses/# of T-unit), CT/T (# of complex T-units/# of T-units), DC/C (# of dependent clauses/# of clauses), DC/T (# of dependent clauses/# of T-units). Amount of coordination: CP/C (# of coordinate phrases/# of clauses), CP/T (# of coordinate phrases/# of T-units), T/S (# of T-units/# of sentences). Degree of phrasal sophistication: CN/C (# of complex nominals/# of clauses), CN/T (# of complex nominals/# of T-units), VP/T (# of verb phrases/# of T-units). Overall sentence complexity: C/S (# of clauses/# of sentences).

These lexical metric in LCA are included as follows: LD(lexical diversity), LS1(Lexical Sophistication-I), LS2(Lexical Sophistication-II), VS1(Verb Sophistication-I), CVS1(Corrected VS1), VS2(Verb Sophistication-II), TTR(Type–Token Ratio), CTTR(Corrected TTR), Root TTR(Root TTR), LogTTR(Bilogarithmic TTR), Uber(Uber Index), LV(Lexical Word Variation). VV1(Verb Variation-I), SVV1(Squared VV1), CVV1(Corrected VV1), VVII(Verb Variation-II), NV(Noun Variation).

The following two tables are the original data on syntactic and lexical complexity by the traditional methods.

**Table A4:** Syntactic complexity for each L2 level in EFCAMDAT2 and the difference between different L2 levels.

Levels	MLS	MLT	MLC	C/S	VP/T	C/T	DC/C	DC/T	T/S	CT/T	CP/T	CP/C	CN/T	CN/C
A1	8.56	8.17	7.04	1.21	1.3	1.16	0.11	0.13	1.05	0.09	0.24	0.2	0.58	0.5
A2	11.59	10.09	7.58	1.53	1.33	1.33	0.21	0.29	1.15	0.23	0.32	0.24	0.76	0.57
B1	13.28	12.01	8.53	1.56	1.41	1.41	0.27	0.37	1.11	0.3	0.3	0.21	1.09	0.78
B2	14.85	13.6	8.93	1.66	1.5	1.52	0.33	0.5	1.09	0.34	0.32	0.21	1.34	0.88
C1	16.32	14.58	9.54	1.71	1.53	1.53	0.33	0.5	1.12	0.34	0.39	0.26	1.56	1.02
A1_C1	7.76	6.41	2.5	0.49	0.23	0.37	0.22	0.37	0.07	0.24	0.16	0.05	0.98	0.52
A2_C1	4.72	4.49	1.96	0.18	0.2	0.2	0.11	0.21	−0.03	0.11	0.08	0.02	0.8	0.45
B1_C1	3.04	2.56	1.01	0.15	0.12	0.12	0.06	0.13	0.01	0.04	0.09	0.04	0.47	0.24
B2_C1	4.72	4.49	1.96	0.18	0.2	0.2	0.11	0.21	−0.03	0.11	0.08	0.02	0.8	0.45
A2_A1	3.04	1.93	0.54	0.31	0.3	0.17	0.1	0.16	0.1	0.13	0.08	0.03	0.17	0.07
B1_A1	4.72	3.85	1.49	0.34	0.53	0.25	0.16	0.25	0.06	0.2	0.06	0.01	0.51	0.27
B2_A1	6.29	5.44	1.89	0.45	0.75	0.36	0.22	0.38	0.04	0.29	0.09	0.01	0.76	0.38
C1_A1	7.76	6.41	2.5	0.49	0.77	0.37	0.22	0.37	0.07	0.28	0.16	0.05	0.98	0.52
A2_A1	3.04	1.93	0.54	0.31	0.3	0.17	0.1	0.16	0.1	0.13	0.08	0.03	0.17	0.07
B1_A2	1.68	1.92	0.95	0.03	0.23	0.08	0.05	0.09	−0.04	0.07	−0.01	−0.02	0.34	0.21
B2_B1	1.57	1.59	0.4	0.11	0.22	0.12	0.07	0.13	−0.01	0.08	0.02	0	0.25	0.1
C1_B2	1.47	0.98	0.61	0.05	0.02	0	0	−0.01	0.03	−0.01	0.07	0.04	0.22	0.14

**Table A5:** Lexical complexity for each L2 level in EFCAMDAT2 and the difference between different L2 levels.

Levels	ld	ls1	ls2	vs1	vs2	cvs1	ttr	msttr	cttr	rttr	logttr	uber	lv	vv1	svv1	cvv1	vv2	nv
A1	0.54	0.56	0.88	0.02	43.42	4.66	0.03	0.73	17.32	24.49	0.74	22.29	0.05	0.02	60.94	5.52	0.01	0.07
A2	0.53	0.52	0.86	0.04	71.49	5.98	0.04	0.75	17.55	24.82	0.75	22.32	0.08	0.04	106.91	7.31	0.01	0.11
B1	0.52	0.47	0.93	0.02	143.27	8.46	0.02	0.79	19.82	28.04	0.72	23.45	0.03	0.02	167.3	9.15	0.01	0.04
B2	0.51	0.48	0.96	0.01	180.81	9.51	0.01	0.79	20.61	29.14	0.71	23.98	0.02	0.01	198.99	9.97	0	0.03
C1_A1	0.52	0.49	0.93	0.02	186.36	9.65	0.02	0.8	20.15	28.5	0.73	23.47	0.03	0.02	218.08	10.44	0.01	0.05
A2_A1	−0.01	−0.04	−0.02	0.02	28.07	1.32	0.01	0.02	0.23	0.33	0.01	0.03	0.03	0.02	45.97	1.79	0	0.04
B1_A1	−0.02	−0.09	0.05	0	99.85	3.8	−0.01	0.06	2.5	3.55	−0.02	1.16	−0.02	0	106.36	3.63	0	−0.03
B2_A1	−0.03	−0.08	0.08	−0.01	137.39	4.85	−0.02	0.06	3.29	4.65	−0.03	1.69	−0.03	−0.01	138.05	4.45	−0.01	−0.04
C1_A1	−0.02	−0.07	0.05	0	142.94	4.99	−0.01	0.07	2.83	4.01	−0.01	1.18	−0.02	0	157.14	4.92	0	−0.02
A2_A1	−0.01	−0.04	−0.02	0.02	28.07	1.32	0.01	0.02	0.23	0.33	0.01	0.03	0.03	0.02	45.97	1.79	0	0.04
B1_A2	−0.01	−0.05	0.07	−0.02	71.78	2.48	−0.02	0.04	2.27	3.22	−0.03	1.13	−0.05	−0.02	60.39	1.84	0	−0.07
B2_B1	−0.01	0.01	0.03	−0.01	37.54	1.05	−0.01	0	0.79	1.1	−0.01	0.53	−0.01	−0.01	31.69	0.82	−0.01	−0.01
C1_B2	0.01	0.01	−0.03	0.01	5.55	0.14	0.01	0.01	−0.46	−0.64	0.02	−0.51	0.01	0.01	19.09	0.47	0.01	0.02
